# The Ventriloquist Illusion as a Tool to Study Multisensory Processing: An Update

**DOI:** 10.3389/fnint.2019.00051

**Published:** 2019-09-12

**Authors:** Patrick Bruns

**Affiliations:** Biological Psychology and Neuropsychology, University of Hamburg, Hamburg, Germany

**Keywords:** cross-modal, multisensory, recalibration, space, ventriloquism

## Abstract

Ventriloquism, the illusion that a voice appears to come from the moving mouth of a puppet rather than from the actual speaker, is one of the classic examples of multisensory processing. In the laboratory, this illusion can be reliably induced by presenting simple meaningless audiovisual stimuli with a spatial discrepancy between the auditory and visual components. Typically, the perceived location of the sound source is biased toward the location of the visual stimulus (the ventriloquism effect). The strength of the visual bias reflects the relative reliability of the visual and auditory inputs as well as prior expectations that the two stimuli originated from the same source. In addition to the ventriloquist illusion, exposure to spatially discrepant audiovisual stimuli results in a subsequent recalibration of unisensory auditory localization (the ventriloquism aftereffect). In the past years, the ventriloquism effect and aftereffect have seen a resurgence as an experimental tool to elucidate basic mechanisms of multisensory integration and learning. For example, recent studies have: (a) revealed top-down influences from the reward and motor systems on cross-modal binding; (b) dissociated recalibration processes operating at different time scales; and (c) identified brain networks involved in the neuronal computations underlying multisensory integration and learning. This mini review article provides a brief overview of established experimental paradigms to measure the ventriloquism effect and aftereffect before summarizing these pathbreaking new advancements. Finally, it is pointed out how the ventriloquism effect and aftereffect could be utilized to address some of the current open questions in the field of multisensory research.

## Introduction

Ventriloquism, literally meaning to speak with the stomach, has a long cultural history that dates back to the ancient Greeks (Connor, 2000). Modern-day ventriloquists entertain their audiences by exploiting the illusion that their voice, produced without overt lip movements, is perceived to originate from the moving lips of a puppet. This visual capture of the perceived auditory location has become one of the most frequently studied examples of multisensory processing in the scientific literature (Stratton, 1897; Klemm, 1909; Thomas, 1941; Jackson, 1953; Thurlow and Jack, 1973; Bertelson and Radeau, 1981; Bertelson and Aschersleben, 1998; Alais and Burr, 2004).

In a typical experimental procedure, participants are presented with a synchronous but spatially discrepant audiovisual stimulus. When asked to localize the sound source, participants usually perceive the auditory stimulus closer to the visual stimulus than it actually is (Bertelson and Radeau, 1981). Although this effect is often tested with simple meaningless stimuli such as tones and light flashes, it has become widely known as the *ventriloquism effect* (Howard and Templeton, 1966). The strength of the ventriloquism effect depends on the relative reliability of the auditory and visual stimuli (Alais and Burr, 2004) as well as on the prior (or expectation) that the two stimuli originated from the same event (Van Wanrooij et al., 2010). This flexible multisensory integration seen at the behavioral level is well-described by Bayesian causal inference models in which the spatial estimates obtained under the assumption of a common vs. separate causes are combined (Körding et al., 2007; Rohe and Noppeney, 2015b). Recent findings suggest that human observers tend to put overly high emphasis on the visual cue in this process (Arnold et al., 2019; Meijer et al., 2019). In addition to the immediate visual influence on auditory localization seen in the ventriloquism effect, exposure to audiovisual stimuli with a consistent audiovisual spatial disparity results in a subsequent recalibration of unisensory auditory spatial perception known as the *ventriloquism aftereffect* (Canon, 1970; Radeau and Bertelson, 1974; Recanzone, 1998). The aftereffect represents an instance of cross-modal learning that can be dissociated from multisensory integration seen in the ventriloquism effect (Bruns et al., 2011a; Zaidel et al., 2011).

The ventriloquism effect and aftereffect are both highly reliable effects that have been replicated in dozens of studies (see Table 1). Both effects are not specific for audiovisual processing but have been demonstrated for audio-tactile and visuo-tactile stimulus pairings as well (Pick et al., 1969; Caclin et al., 2002; Bruns and Röder, 2010; Bruns et al., 2011b; Samad and Shams, 2016, 2018). This robustness and versatility make them ideal experimental paradigms to study basic mechanisms of multisensory integration and learning. The extensive literature on the ventriloquism effect and aftereffect has been summarized in several excellent reviews (Bertelson and de Gelder, 2004; Woods and Recanzone, 2004; Recanzone, 2009; Chen and Vroomen, 2013). However, since the last comprehensive review by Chen and Vroomen (2013), several new lines of research have emerged that have helped clarifying the role of the reward and motor systems in cross-modal binding, the time scales involved in recalibration, and the neural mechanisms underlying multisensory integration and learning. The aim of the present review article is to provide an update on these exciting recent developments which are summarized in Table 1. In addition, the following section describes some of the standard procedures to measure the ventriloquism effect and aftereffect to encourage more researchers to utilize these effects in their quest to tackle the remaining open questions in multisensory research.

**Table 1:** Key studies on the ventriloquism effect and aftereffect published since 2013.

Study	Main finding
Arnold et al. (2019) and Meijer et al. (2019)	Visual bias in VE is stronger than predicted by maximum likelihood integration
Bruns et al. (2014)	Monetary reward for accurate sound localization reduces the VE
Zierul et al. (2019)	Reduced VE for self-initiated audiovisual stimuli
Zaidel et al. (2013)	Feedback results in yoked recalibration of both cues in the same direction
Pages and Groh (2013)	VAE depends on visual feedback rather than on audiovisual synchrony
Berger and Ehrsson (2013) and Berger and Ehrsson (2018)	Imagined visual stimuli induce a VE and VAE
Delong et al. (2018)	Subliminal visual stimuli induce a (reduced) VE
Bruns and Röder (2015)	Immediate and cumulative VAE are dissociable processes
Bosen et al. (2017)	VAE accumulates with repetitions and decays over time
Bosen et al. (2018)	VAE consists of both a large and transient initial localization shift, as well as a smaller and more enduring shift
Mendonça et al. (2015)	Last audiovisual trial affects subsequent VAE the most
Watson et al. (2019)	VAE involves distinct recalibration mechanisms operating at different time scales
Bruns and Röder (2019)	Repeated training sessions enhance the VAE over days
Callan et al. (2015)	VE is associated with modulation of activity in space-sensitive auditory cortex
Bonath et al. (2014)	Separate but adjacent auditory regions code VE to synchronous and asynchronous stimuli
Rohe and Noppeney (2015a) and Rohe and Noppeney (2016)	Multisensory integration and causal inference are performed in parietal regions
Aller and Noppeney (2019)	Causal inference in the brain is accomplished by a dynamic encoding of multiple spatial estimates
Park and Kayser (2019)	VE and immediate VAE have a common neural substrate in parietal cortex
Cuppini et al. (2017)	Biologically inspired neural network model explains behavioral VE
Zierul et al. (2017)	VAE results in persistent adjustments of spatial representations in auditory cortex
Bruns and Röder (2017)	VAE depends on the sensory context
Odegaard et al. (2017)	Cross-modal binding (i.e., VE) increases after exposure to synchronous but spatially unrelated stimuli
Odegaard and Shams (2016)	Cross-modal binding (i.e., VE) is stable over time in adulthood

*VE, ventriloquism effect; VAE, ventriloquism aftereffect*.

## Measuring the Ventriloquism Effect and Aftereffect

The ventriloquism effect and aftereffect have been reliably obtained with a large variety of different localization tasks. These tasks can be categorized into absolute (or continuous) localization measures and relative (or dichotomous) localization measures. In absolute localization tasks, participants directly localize the stimuli with a hand pointer (Lewald, 2002; Bruns and Röder, 2015, 2017, 2019) or by performing a finger (Frissen et al., 2003, 2005, 2012), head (Recanzone, 1998; Van Wanrooij et al., 2010), or eye movement (Kopco et al., 2009; Pages and Groh, 2013) toward the perceived stimulus location. Some studies have used categorical responses (e.g., left, center, or right) instead (Bonath et al., 2007, 2014; Bruns and Röder, 2010; Bruns et al., 2011a; Rohe and Noppeney, 2015a, 2016; Zierul et al., 2017). While categorical responses are less sensitive than continuous measures, they are preferable in studies involving electrophysiological or neuroimaging recordings to reduce motor noise. An alternative are relative localization tasks, in which stimulus location is judged relative to central fixation (i.e., left vs. right) or relative to a reference stimulus in a two-alternative forced choice (2AFC) manner (Bertelson and Aschersleben, 1998; Recanzone, 1998; Bruns et al., 2011b; Berger and Ehrsson, 2018). Some authors have also advocated two-interval forced choice (2IFC) procedures because they are less susceptible to response strategies (Alais and Burr, 2004; Vroomen and Stekelenburg, 2014).

**Figure 1:**
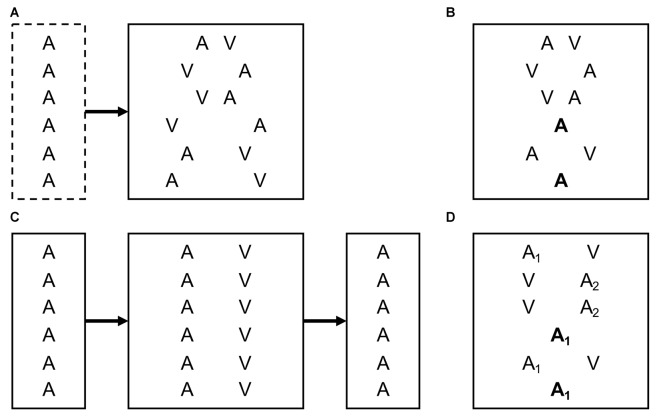
Typical experimental designs to measure the ventriloquism effect and aftereffect. Exemplarily, letters indicate unimodal auditory (A) trials and relative locations of auditory (A) and visual (V) stimuli in bimodal trials. In an actual experiment, absolute stimulus locations typically vary between trials. **(A)** Ventriloquism effect. Participants have to localize cross-modal stimuli with varying spatial discrepancies. Unisensory localization is assessed in an optional pretest block. Comparison of responses between equivalent left- and right-side discrepancies or between bimodal and unimodal stimuli reveal the size of the ventriloquism effect. **(B)** Immediate ventriloquism aftereffect. Intermixed presentation of bimodal and unimodal trials. Localization in unimodal trials is modulated by the cross-modal discrepancy in the directly preceding bimodal trial. **(C)** Cumulative ventriloquism aftereffect. Unisensory sound localization is measured before and after exposure to cross-modal stimuli with a consistent spatial discrepancy. **(D)** Design used in Bruns and Röder (2015) to measure the immediate and cumulative ventriloquism aftereffects concurrently. Tones of two different sound-frequencies (A_1_ and A_2_) are consistently paired with opposite directions of cross-modal spatial discrepancy. Differences in localization responses between unimodal trials preceded by audiovisual trials with leftward vs. rightward discrepancy reveal the immediate aftereffect, and differences between unisensory localization of A_1_ vs. A_2_ reveal the cumulative aftereffect (see text for details).

The study design differs slightly depending on whether the ventriloquism effect or the ventriloquism aftereffect (or both) are to be measured (see Figure 1). To measure the ventriloquism effect, it is critical that different degrees and directions of cross-modal spatial disparity are presented in a random order to avoid cumulative recalibration effects during the test block (Bertelson and Radeau, 1981; Bertelson and de Gelder, 2004). In addition, baseline localization can be assessed in unimodal trials, either intermixed with the bimodal trials or in a separate pretest block. Aside from the size of the localization bias in the bimodal trials, the ventriloquism effect has been conceptualized as the percentage of trials in which participants perceive the (spatially disparate) cross-modal stimuli as originating from a common cause or the same location (Chen and Spence, 2017). Localization bias and perception of unity are usually correlated (Hairston et al., 2003; Wallace et al., 2004) but measure different aspects of cross-modal integration (Bertelson and Radeau, 1981; Bosen et al., 2016; Chen and Spence, 2017).

When assessing the ventriloquism aftereffect, a distinction needs to be made between immediate and cumulative recalibration effects (Bruns and Röder, 2015). In a study design in which unimodal trials are intermixed with bimodal trials (see Figure 1B), Wozny and Shams (2011) showed that localization responses in unimodal trials are systematically influenced by the cross-modal spatial disparity in the directly preceding bimodal trial, indicating an immediate or trial-by-trial recalibration effect. By contrast, the cumulative ventriloquism aftereffect requires exposure to a consistent cross-modal disparity (e.g., visual stimuli always 10° to the right of auditory stimuli). Typically, unisensory sound localization is measured before and after the exposure block (see Figure 1C), and the cumulative aftereffect is revealed by a shift in unisensory localization from pre- to post-test (Recanzone, 1998; Lewald, 2002; Frissen et al., 2003; Bruns and Röder, 2017).

Bruns and Röder (2015) recently introduced a procedure that allows assessing both immediate and cumulative aftereffects (as well as ventriloquism effects) at the same time (see Figure 1D). In this paradigm, auditory-only and audiovisual trials were intermixed. Crucially, tones of two different sound frequencies were used that were paired with opposite directions of audiovisual disparity (leftward vs. rightward). Sound localization responses in auditory-only trials (averaged across tone frequencies) were modulated by the direction of audiovisual disparity in the directly preceding audiovisual trial, indicating an immediate aftereffect. Additionally, sound localization responses differed between the two tone-frequencies, indicating a frequency-specific cumulative aftereffect induced by the consistent pairing of tone-frequency and direction of audiovisual disparity (but see Frissen et al., 2003, 2005; Bruns and Röder, 2017; for a discussion of the sound frequency specificity of the cumulative aftereffect).

## Recent Findings

### Top-Down Influences on Cross-Modal Binding and Learning

A long-standing debate in multisensory research is the extent to which multisensory processing is influenced by top-down factors (Röder and Büchel, 2009; Talsma et al., 2010). Contrary to earlier findings suggesting that the ventriloquism effect and aftereffect reflect largely automatic processes (Bertelson et al., 2000; Vroomen et al., 2001; Passamonti et al., 2009; Odegaard et al., 2016), several recent lines of evidence have identified top-down influences on the ventriloquism effect and aftereffect.

In a study by Bruns et al. (2014), participants could earn either a high or a low monetary reward for accurate sound localization performance, which put their motivational goal of maximizing the reward in conflict with the auditory spatial bias induced by the ventriloquism effect. As compared to stimuli associated with a low reward, the ventriloquism effect was significantly reduced for high reward stimuli. A similar reduction of the ventriloquism effect was observed when emotionally salient auditory stimuli (fearful voices) were presented prior to the audiovisual test phase (Maiworm et al., 2012). In both cases, the experimental manipulations did not affect unisensory auditory localization performance, suggesting that top-down influences from the emotion and reward systems specifically reduced cross-modal binding. A similar pattern of results was observed in a recent study in which participants either actively initiated audiovisual stimulus presentations with a button press or were passively exposed to the same stimuli. Contrary to the intuitive assumption that self-initiation would increase the prior expectation that auditory and visual stimuli had a common cause, a reduction of the size of the ventriloquism effect was observed for self-initiated stimuli, possibly due to an increased sensitivity to cross-modal spatial discrepancies in the self-initiation condition (Zierul et al., 2019).

A second line of research investigated the effects of feedback information about the stimulus location on cross-modal recalibration. In a visuo-vestibular version of the ventriloquism aftereffect, participants received a reward for correct localization responses which was contingent either on the visual or on the vestibular cue. This manipulation resulted in a yoked recalibration of both cues in the same direction (Zaidel et al., 2013), whereas passive exposure without feedback shifted both cues independently toward each other (Zaidel et al., 2011). The importance of feedback information was substantiated in the classic audiovisual ventriloquism aftereffect. Here, asynchronous stimuli in which the visual stimulus lagged the auditory stimulus and, thus, provided feedback about the auditory location were more effective in inducing an aftereffect than synchronous stimuli in which the visual stimulus was extinguished too quickly to provide feedback (Pages and Groh, 2013). Thus, feedback, which presumably exerts top-down influences on perception, might be an important but previously overlooked driver of cross-modal recalibration.

Finally, in a third line of research, Berger and Ehrsson (2013, 2014, 2018) showed that imagining a visual stimulus at a location discrepant to an auditory stimulus had the same effect on auditory localization as actually seeing a visual stimulus at that location. Both imagery-induced ventriloquism effects (Berger and Ehrsson, 2013, 2014) and aftereffects (Berger and Ehrsson, 2018) were obtained. Explicit mental images were, thus, integrated with auditory sensory input in a similar manner as actual visual input, providing strong evidence for top-down influences on multisensory processing. A somewhat opposite approach was taken by Delong et al. (2018), who used continuous flash suppression to render an actual visual stimulus invisible. They obtained a significant ventriloquism effect with the invisible stimuli, which was, however, reduced in size compared to visible stimuli. Taken together, these results show that the ventriloquism effect is influenced by both bottom-up and top-down processes.

### Time Scales of Cross-Modal Recalibration

Cross-modal recalibration in the ventriloquism aftereffect has been described at two different time scales. Initial studies measured shifts in sound localization after exposure to several hundred audiovisual trials with a consistent spatial disparity (Radeau and Bertelson, 1974; Recanzone, 1998; Lewald, 2002), implicitly assuming that recalibration requires accumulated evidence of cross-modal mismatch. This assumption was challenged by findings demonstrating immediate effects on sound localization after a single audiovisual exposure stimulus (Wozny and Shams, 2011). Several recent studies have addressed the theoretically important question of how immediate and cumulative cross-modal recalibration are related.

A consistent finding is that the size of the ventriloquism aftereffect increases if several audiovisual exposure trials with a consistent spatial disparity precede the auditory test trials (Wozny and Shams, 2011; Bruns and Röder, 2015; Bosen et al., 2017, 2018), until the aftereffect reaches a maximum after about 180 exposure trials (Frissen et al., 2012). The last audiovisual stimulus, however, seems to have a particularly strong influence on subsequent sound localization (Mendonça et al., 2015). Theoretically, the immediate and cumulative portions of the ventriloquism aftereffect could be explained by the same underlying mechanism, a strong but rapidly decaying immediate aftereffect with a long tail that allows for accumulation across trials (Bosen et al., 2018). However, recent experimental evidence suggests dissociable mechanisms underlying immediate and cumulative recalibration (Bruns and Röder, 2015; Watson et al., 2019).

A controversial point is the longevity of the (cumulative) ventriloquism aftereffect after cessation of cross-modal discrepancy training. While some studies observed a rapid decay of the aftereffect if there was a delay between audiovisual exposure and auditory localization posttest (Bosen et al., 2017, 2018), others have found no significant decline of the aftereffect (Frissen et al., 2012). However, it was assumed that the aftereffect would last at most until new (spatially coincident) audiovisual evidence is encountered, as would naturally occur after leaving the experimental situation (Recanzone, 1998). Contrary to this assumption, a recent study showed that repeated exposure to audiovisual stimuli with a consistent spatial disparity enhanced the ventriloquism aftereffect over the course of several days, that is, aftereffects were still present after 24 h and accumulated with additional audiovisual discrepancy training (Bruns and Röder, 2019). This finding raises the possibility that cross-modal recalibration effects are context-specific (e.g., for the laboratory situation), making them more stable than previously thought.

### Neural Mechanisms Underlying Cross-Modal Binding and Learning

Neuroimaging studies have shown that the ventriloquism effect is associated with a modulation of activity in space-sensitive regions of the planum temporale in auditory cortex (Bonath et al., 2007, 2014; Callan et al., 2015; Zierul et al., 2017). Behaviorally, the ventriloquism effect is reduced if audiovisual stimuli are presented asynchronously (Slutsky and Recanzone, 2001; Wallace et al., 2004). Interestingly, Bonath et al. (2014) showed that separate (but adjacent) regions of the planum temporale coded ventriloquist illusions to synchronous and asynchronous audiovisual stimuli, which might suggest an involvement of different multisensory temporal integration windows.

Adjustments of auditory spatial processing in the ventriloquism effect have been linked to feedback influences on auditory cortex activity (Bonath et al., 2007; Bruns and Röder, 2010). Recent EEG and functional magnetic resonance imaging (fMRI) evidence has indeed implicated multisensory association areas of the intraparietal sulcus in the generation of the ventriloquism effect. While primary sensory areas initially encoded the unisensory location estimates, posterior intraparietal sulcus activity reflected the integrated estimate which depends on the relative reliabilities of the auditory and visual estimates (Rohe and Noppeney, 2015a). The brain needs to weigh the unisensory estimate against the integrated estimate due to the inherent uncertainty about the true causal structure (Körding et al., 2007), and this weighing was reflected in anterior intraparietal sulcus activity emerging from 200 ms poststimulus onwards (Rohe and Noppeney, 2015a; Aller and Noppeney, 2019). Parietal representations were found to mediate both multisensory integration and the immediate recalibration of unisensory perception in the subsequent auditory trial (Park and Kayser, 2019). In a re-analysis of their data, Rohe and Noppeney (2016) further showed that parietal areas take into account top-down task relevance (i.e., which modality had to be reported), which might suggest a neural basis for other top-down influences discussed in the subsection “Top-Down Influences on Cross-Modal Binding and Learning.” EEG and MEG studies have revealed a crucial role of neural oscillations in orchestrating the interplay between stimulus-driven and top-down effects in multisensory processing (Senkowski et al., 2008; Keil and Senkowski, 2018). Based on the available evidence, neural network models of the ventriloquism effect have been developed (Magosso et al., 2012; Cuppini et al., 2017).

While the neural computations underlying multisensory spatial integration and immediate recalibration might critically depend on parietal areas, cross-modal recalibration in the cumulative ventriloquism aftereffect was found to result in an enduring change of spatial representations in the planum temporale and an increase of connectivity between the planum temporale and parietal areas (Zierul et al., 2017). This suggests that sustained changes in unisensory sound localization reflect altered bottom-up processing along the auditory “where” pathway (Bruns et al., 2011a).

## Future Directions

The ventriloquism effect and aftereffect have generated an abundance of new insights into the mechanisms of multisensory processing in recent years. Future challenges include translating these new findings into a more general theoretical framework of multisensory processing in naturalistic environments as well as clarifying the developmental trajectory of multisensory spatial integration and learning.

In real-world scenarios, cross-modal stimuli are usually accompanied by a myriad of other continuously changing stimuli. This sensory context inevitably modulates how a particular stimulus is processed (Bruns and Röder, 2017; Bruns and Watanabe, 2019) and shapes priors for processing that stimulus during future encounters (Habets et al., 2017; Odegaard et al., 2017). In addition, the sensory evidence itself might be corrupted by varying amounts of noise. Interestingly, in a phenomenon referred to as cross-modal stochastic resonance, it has been found that intermediate levels of noise in one sensory modality can enhance (rather than impair) responses to weak stimuli in another sensory modality (Manjarrez et al., 2007; Mendez-Balbuena et al., 2018). Future studies should address how learned priors and sensory context interact with bottom-up sensory evidence in the brain. To address these questions, emerging technologies like augmented and virtual reality might help bringing the ventriloquism effect and aftereffect paradigm closer to more complex real-world scenarios (Sarlat et al., 2006;Kytö et al., 2015).

Multisensory spatial processing appears relatively stable over time during adulthood (Odegaard and Shams, 2016), but surprisingly few studies have tested its ontogenetic development in humans. Non-human animal studies have typically investigated visual calibration of auditory spatial representations over rather long time scales of weeks to months (King, 2009), but the developmental trajectory of short-term recalibration effects (as observed in the ventriloquism aftereffect) and its relation to optimal cross-modal integration (as measured in the ventriloquism effect) remains unknown. To assess developmental influences on multisensory spatial functions, retrospective studies in which the impact of sensory deprivation during sensitive periods of development (e.g., due to blindness) is tested in adult individuals are needed as well (Occelli et al., 2012).

With their long history, the ventriloquism effect and aftereffect are timeless experimental paradigms and invaluable tools for the field of multisensory research. Hopefully, this review article will stimulate further discoveries in the years to come.

## Author Contributions

The author confirms being the sole contributor of this work and has approved it for publication.

## Conflict of Interest Statement

The author declares that the research was conducted in the absence of any commercial or financial relationships that could be construed as a potential conflict of interest.
